# Individual and institutional determinants of caesarean section in referral hospitals in Senegal and Mali: a cross-sectional epidemiological survey

**DOI:** 10.1186/1471-2393-12-114

**Published:** 2012-10-22

**Authors:** Valérie Briand, Alexandre Dumont, Michal Abrahamowicz, Mamadou Traore, Laurence Watier, Pierre Fournier

**Affiliations:** 1 Research Centre of CHU Sainte-Justine, Montreal, Canada; 2 Institut de Recherche pour le Développement, UMR216, Faculté de Pharmacie, laboratoire de parasitologie, 4, avenue de l’Observatoire, 75005, Paris, France; 3 UMR 216, Paris Descartes University, Sorbonne Paris Cité, Faculté de Pharmacie, Paris, France; 4 Department of Epidemiology and Biostatistics, McGill University, Montreal, Canada; 5 Referral health center of the Commune V, Bamako, Mali; 6 National Institute for Medical Research U657, Paris, 75015, France; 7 Pasteur Institute, Pharmacoepidemiology and Infectious Diseases unit, Paris, France; 8 Université Versailles Saint Quentin, EA4499, Garches, 92380, France; 9 CRCHUM Research Centre, University of Montreal, Montreal, Canada

**Keywords:** Caesarean section, Africa, Epidemiology

## Abstract

**Background:**

Two years after implementing the free-CS policy, we assessed the non-financial factors associated with caesarean section (CS) in women managed by referral hospitals in Senegal and Mali.

**Methods:**

We conducted a cross-sectional survey nested in a cluster trial (QUARITE trial) in 41 referral hospitals in Senegal and Mali (10/01/2007–10/01/2008). Data were collected regarding women’s characteristics and on available institutional resources. Individual and institutional factors independently associated with emergency (before labour), intrapartum and elective CS were determined using a hierarchical logistic mixed model.

**Results:**

Among 86 505 women, 14% delivered by intrapartum CS, 3% by emergency CS and 2% by elective CS. For intrapartum, emergency and elective CS, the main maternal risk factors were, respectively: previous CS, referral from another facility and suspected cephalopelvic-disproportion (adjusted Odds Ratios from 2.8 to 8.9); vaginal bleeding near full term, hypertensive disorders, previous CS and premature rupture of membranes (adjusted ORs from 3.9 to 10.2); previous CS (adjusted OR=19.2 [17.2-21.6]). Access to adult and neonatal intensive care, a 24-h/day anaesthetist and number of annual deliveries per hospital were independent factors that affected CS rates according to degree of urgency. The presence of obstetricians and/or medical-anaesthetists was associated with an increased risk of elective CS (adjusted ORs [95%CI] = 4.8 [2.6-8.8] to 9.4 [5.1-17.1]).

**Conclusions:**

We confirm the significant effect of well-known maternal risk factors affecting the mode of delivery. Available resources at the institutional level and the degree of urgency of CS should be taken into account in analysing CS rates in this context.

## Background

The worldwide rise in caesarean section (CS) rates has become a major public-health concern due to potential maternal and perinatal risks, cost issues and inequity of access [1-4]. The increased CS rates observed in many developed and middle-income countries contrast with the very low rates in numerous low-resource settings. According to recent data, in sub-Saharan Africa (SSA), only 3% of all deliveries occur by CS [3], compared to 24% in North America and 31% in Central America [2].

In order to improve access to emergency obstetric care (EmOC), national free-CS policies are being trialled in several SSA countries [5]. In Senegal, exemption of CS fees was introduced in 2005 in all referral hospitals of the five poorest areas. The policy was then extended to referral hospitals in other areas (excluding Dakar) [6,7]. In Mali, the free-CS policy was adopted on a national basis in 2006. In both countries, government funded schemes provide CS kits with basic supplies to district hospitals and reimburse regional hospitals for lost caesarean revenues. In Mali, fee exemption is for surgery, CS supplies, drugs and hospitalization, whereas only supplies are free in Senegal.

In addition to free-CS policies, both governments have increased comprehensive EmOC services (including caesarean section), which are now available according to United Nations standards [8]. Because of the scarcity of skilled health professionals, general practitioners and nurses have been trained in emergency obstetric surgery and anaesthesia since 1998 [9].

The few studies that have assessed the effectiveness of fee exemption for pregnant women have shown increased use of maternal-health services, with higher rates of in-hospital deliveries and earlier antenatal-care visits [10]. In Senegal, Witter et al. found that facility-based CS rates had increased by 130% 1 year after implementation of free CS [6,7]. However, there is little evidence that this rapid increase is justified. Several studies have identified factors associated with increased CS, but independent prognostic factors vary because of differences in patient populations, in the variables collected and in statistical methods. Also, few studies have been conducted in developing countries [11-14]; thus, it remains difficult to specify determinants for CS in SSA and to propose a CS classification that facilitates international and local comparisons.

Herein, we have assessed the non-financial factors associated with the likelihood of women receiving a CS when managed by referral hospitals in Senegal and Mali 2 years after implementation of the free-CS policy. We have assumed that this policy was fully in place before the beginning of our study. Because financial barriers were *a priori* overlapped, we also hypothesized that facility-based CS rates mainly depended on women’s and hospitals’ characteristics.

## Methods

This secondary analysis included data extracted from a cluster-randomized controlled trial (QUARITE trial) in referral hospitals in Senegal and Mali. The protocol of the trial was approved by the ethics committee of Sainte-Justine Hospital in Montreal, Canada, and by the national ethics committees in Senegal and in Mali. The study protocol of the QUARITE trial and data collection procedures have already been published [15]. Briefly, the trial aimed to assess the effectiveness of the multifaceted Advances in Labour and Risk Management (ALARM) International Program – based on maternal death reviews – to reduce maternal mortality. Secondary goals included evaluation of the relationships between effectiveness and resource availability, service organization, medical practices that included CS rates, and satisfaction among health personnel.

### Study site and population

The trial was conducted in 46 out of a total of 49 eligible referral hospitals – 26 in Senegal and 23 in Mali – spread across both countries. A hospital was eligible for the trial if it had functional operating rooms and carried out >800 deliveries annually. Three eligible hospitals were excluded for the trial: two already had a structured programme for carrying out maternal-death audits before the project began, and the other hospital did not receive written consent from the local authorities.

**Figure 1:**
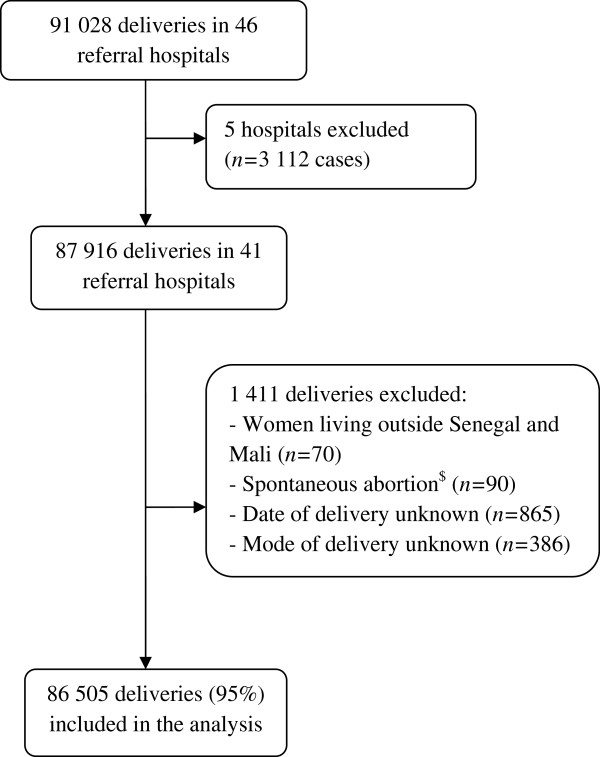
**Flow chart. A total of 91,028 women delivered in the 46 referral hospitals selected for the QUARITE trial during the first year of the trial (from October 2007 to October 2008).** Five hospitals were excluded from the analysis: four did not carry out any caesarean deliveries during the study period and one had data from mid-2008 only. ^$^ Spontaneaous abortion was defined as birth weight less than 500 grams.

For the current analysis, we used the data collected during the first year of the trial – from October 2007 to October 2008 – when the ALARM intervention had not yet been implemented (i.e. pre-intervention phase of the trial). Therefore, there were no constraints or guidelines regarding investigations, treatments, admission and discharge decisions. Five hospitals out of the 46 included in the trial were excluded because four did not carry out any CS during the study period, and one only had data from mid-2008 (Figure 1). All women who delivered in the 41 centres during the study period were included in the analyses, except those who lived outside Senegal or Mali, had a spontaneous abortion, and if the delivery date or mode of delivery was unknown.

### Data collection

Trained midwives who were supervised by the national coordinators of the survey collected data from medical records. In each country, data were collected on a daily basis on every woman who gave birth in every selected facility. It included: maternal demographic characteristics, obstetric history, prenatal care, management of labour and delivery, complications, and the vital status of both mother and child until hospital discharge. Pathologies during the current pregnancy and CS indications were reported using open questions and a pre-defined list of diagnoses or CS indications. The national coordinators of the study regularly verified that data collection was exhaustive (by comparing the number of eligible patients on the hospital’s birth register with the number of forms collected) and also checked data quality in a random sample of forms [15]. Between October 2007 and October 2008, 99% of the eligible women were included in the trial. The concordance rate – defined as the proportion of patient forms whose information was concordant with the hospital registers and medical records – was of 88% during the study period. Missing data for all variables accounted for <1% of cases, except for oxytocin use, which was missing for 5% of cases.

For each institution, available resources were recorded in September 2007 and October 2008. A standardized inventory, developed by Villar [16], based on the WHO’s Complexity Index was used. This reflects the availability of different categories of resources required to provide high quality emergency obstetric care: basic services, screening tests, basic emergency obstetric resources, intrapartum care, general medical services, anaesthesiology resources, human resources, academic resources, and clinical protocols. Because resources changed during the study period, we split the study into period 1, from October 2007 to March 2008, and period 2, from April to October 2008. Women who delivered during periods 1 and 2 were assumed to have access to resources recorded in the first and second inventories, respectively. Regarding human resources, we created a categorical variable to distinguish between four different levels based on the number and qualifications of the medical staff: level I (‘reference’ group): general practitioners (GPs) trained in obstetrics, with nurse-anaesthetist(s) and two or less midwives; level II: trained GP(s), with nurse-anaesthetist(s) and three or more midwives; level III: at least one obstetric/gynaecology specialist, +/− trained GP(s), with nurse-anaesthetist(s) and three or more midwives; level IV: at least one obstetrics/gynaecology specialist, +/− trained GP(s), with at least one medical anaesthetist and three or more midwives.

### Statistical analyses

Mode of delivery was the main outcome of interest. Because the factors associated with CS differed according to the degree of urgency [14], we performed three distinct analyses, i.e. (i) emergency CS before labour (referred to as “emergency”) vs. all other deliveries, (ii) emergency intrapartum CS (“intrapartum”) vs. all vaginal deliveries, and (iii) elective CS vs. all delivery births with a trial of labour, which included both vaginal and intrapartum caesarean deliveries. No distinction was made between spontaneous vs. operative vaginal deliveries.

For each type of CS, analysis was performed using a two-step procedure. As the first step, we assessed only individual factors, as they were expected to have the highest impact on CS likelihood. Potential individual risk factors were selected according to results obtained from previous studies in low- and middle-income countries [11,16-19]: age, parity, previous CS, multiple pregnancy (vs. single pregnancy), hypertensive disorders, vaginal bleeding near full term, suspected cephalopelvic-disproportion, suspected intrauterine death, premature rupture of the membranes, referral from another hospital, premature labour and oxytocin use. We considered that women did not have a condition if it had not been reported by a midwife. Obstetric complications that occurred during labour (i.e. obstructed labour or foetal distress) were not included in the analyses because they closely affected the decision regarding CS. Referral from another hospital was considered as a potential marker for more severe conditions because of delays due to large travel distances or lack of transportation. Both tri-variate (i.e., adjusted for the country and the period) and multi-variable analyses were performed. All variables, regardless of their association with CS in tri-variate analyses, were included in the multivariable model. They were all kept in the final model as they were independent and highly significant determinants of outcome (*P*<0.01). We used a conservative significance level to account for multiple analyses, and a very large sample size implied that any clinically relevant association was very significant.

As the second step of analyses, we assessed which institutional factors were independently associated with CS, while adjusting for individual factors that were selected into the final multivariable model of the first step. Institutional factors considered for analysis were all items recorded in the standardized inventories (see the list of factors in Additional file 1). We did not use the Complexity Index, which aggregates the information on all available resources, but we tested each factor to determine which specifically influenced the decision for CS. Then, as in step one, all variables, regardless of their association with CS in tri-variate analysis, were considered for the multivariable analysis. In the final model, only those variables with a *P*<0.01, after a forward-stepwise procedure, were selected. We used a forward elimination procedure to account for very high sample size and high correlation between institutional variables. The level of qualification of the medical staff and the time period were forced into the final multivariable model.

We used a logistic mixed model to account for the dependence of observations within hospital [20]. Indeed, including a random intercept to the model, assumed that women who delivered in the same hospital were more likely to have the same mode of delivery – because of common individual characteristics and shared institutional resources – than women who delivered in different hospitals. Also, we estimated the relative contribution of individual and institutional factors to the variability of each outcome (i.e., elective, emergency and intrapartum CS) between hospitals. In that purpose, we used the ratios of the random intercept variances [21].

To determine the effect of medical-staff configuration, we calculated the variation of elective CS rates between hospitals in women with either a low risk for CS (primiparous, <35 years old, with singleton pregnancy and no hypertensive disorders) or a high risk for CS (multiparous, >35 years old, with previous caesarean section and hypertensive disorders) in level I and IV hospitals [22].

All statistical analyses were performed using SAS system software (SAS Institute Inc., Cary, NC, USA). Hierarchical logistic mixed-regression models were estimated using the PROC NLMIXED procedure.

## Results

**Figure 2:**
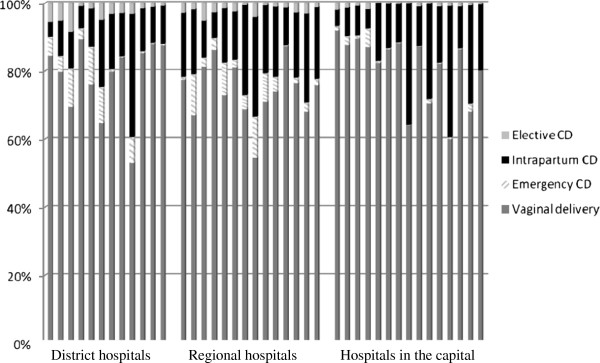
**Proportions of vaginal deliveries (both spontaneous and instrumental), emergency, intrapartum and elective caesarean sections in each study hospital and according to the type of hospital (district hospitals, regional hospitals and hospitals in the capital).** The proportions of elective, emergency and intrapartum caesarean sections varied between hospitals, ranging from 0–8.3% (median: 1.3%), 0–12% (1.9%), and 4.5–38.7% (14.4%) of all deliveries, respectively.

A total of 86 505 women were included in the analyses (Table 1). CS accounted for 19.8% (95% CI: 19.4–20) of all deliveries, with a higher rate in Senegal (20.9%, 95% CI: 20.5–21.3) than in Mali (18.5%, 95% CI: 18.1–18.8). The majority of CS involved intrapartum delivery (73%), whereas emergency and elective CS represented 16% and 11%, respectively. These proportions varied considerably between hospitals (Figure 2). In contrast, the proportion of operative vaginal deliveries accounted for only 2% of deliveries. Maternal indications accounted for 77% of all CS (Table 2). The most commonly reported indications for elective, emergency and intrapartum CS were, respectively, previous caesarean section (38%), hypertensive disorders (27%) and prolonged/obstructed labour (37%).

**Table 1:** **Women’ characteristics, by time period,*n*(%) (Senegal and Mali, October 2007-October 2008)**

	**Period 1** **(*n*=45 261)**	**Period 2 ** **(*n*=41 244)**	**All women ** **(*n=*86 505)**
Age ≥35 years	6 633 (15)	5 704 (14)	12 337 (14)
Nulliparous	16 319 (36)	13 769 (33)	30 088 (35)
Previous caesarean section	3 119 (7)	3 082 (7)	6 201 (7)
**Current pregnancy**			
Multiple pregnancy	1 640 (4)	1 759 (4)	3 399 (4)
Hypertensive disorders*	3 197 (7)	2 874 (7)	6 071 (7)
Vaginal bleeding (near full term)	1 497 (3)	1 616 (4)	3 113 (4)
Suspected cephalopelvic-disproportion^**^	99 (0.2)	114 (0.3)	213 (0.2)
Suspected intrauterine death	844 (2)	696 (2)	1 540 (2)
Premature rupture of the membranes	1 805 (4)	1 278 (3)	3 083 (4)
Referral from another hospital	11 021 (24)	10 579 (26)	21 600 (25)
**Labour**			
Premature labour	620 (1)	625 (1)	1 245 (1)
Oxytocin use	1 321 (3)	1 180 (3)	2 501 (3)
**Mode of delivery**			
*Vaginal delivery*			
Spontaneous	35 756 (79)	31 857 (77)	67 613 (79)
Operative	920 (2)	914 (2)	1 834 (2)
*Caesarean section*^*£*^			
Emergency	1 438 (3)	1 233 (3)	2 671 (3)
Intrapartum	6 044 (13)	6 382 (16)	12 446 (14)
Elective	1 083 (3)	858 (2)	1 941 (2)

^*^Chronic hypertension, gestational hypertension, pre-eclampsia, eclampsia or HELLP syndrome. ^**^Suspected cephalopelvic-disproportion reported as “excessive fundal-height” or “pathologic pelvis”. ^£^CS accounted for 19.8% (95% CI: 19.4–20.0) of all deliveries, with a higher rate in Senegal (20.9%, 95% CI: 20.5–21.3) than in Mali (18.5%, 95% CI: 18.1–18.8). The majority of CS involved intrapartum delivery (73%), whereas emergency and elective CS represented 16% and 11%, respectively.

**Table 2:** Main reported indications for caesarean section according to the type of CS

	**Elective (*n*=1 872)**	**Emergency (*n*=2 623)**	**Intrapartum (*n*=12 172)**	**Total (*n*=16 667)**
**Maternal indications** n (%)				**12 753 (77)**
Prolonged/obstructed labour or suspected cephalopelvic disproportion	301 (16)	197 (8)	4 451 (37)	4 949 (30)
Previous caesarean section	712 (38)	320 (12)	1 209 (10)	2 241 (13)
Pre-eclampsia/eclampsia	96 (5)	717 (27)	497 (4)	1 310 (8)
Abruptio placentae	*n*=4	422 (16)	685 (6)	1 111 (7)
Uterine rupture	*n*=0	12 (1)	755 (6)	767 (5)
Placenta praevia	28 (2)	216 (8)	429 (3)	673 (4)
Other^*^	491 (26)	489 (19)	722 (6)	1 702 (10)
**Foetal indications** n (%)				**3 914 (23)**
Foetal distress	45 (2)	110 (4)	1 952 (16)	2 107 (13)
Non-cephalic presentation	83 (5)	54 (2)	1 143 (9)	1 280 (8)
Other^**^	112 (6)	86 (3)	329 (3)	527 (2)

^*^ Other maternal indications: post-term (1.3%), maternal request (0.7%), vaginal bleeding near full term (0.6%), post-mortem (0.3%), vaginal fistula (0.2%), genital infection (0.1%), HIV-infection (n=2), caesarean section on maternal demand (0.1%), premature rupture of membranes (*n*=5), not specified (7%); ** Other foetal indications: suspected intrauterine growth retardation (0.3%), not specified(2%).

The scarcest institutional resources were those related to foetal monitoring, neonatal care and advanced screening tests (see Additional file 1). Among hospitals located in the capital and regional hospitals, there was at least one obstetric specialist (i.e., level III and IV hospitals). In contrast, in district hospitals, two-thirds had trained GPs only (i.e. level I and II hospitals).

Individual and institutional factors related to each type of CS are shown in Table 3. Because no differences in factor effects between periods were observed, the results are presented for the total study period. Only results for multivariable analyses are presented. Previous caesarean section was strongly associated with CS, and particularly with elective CS. The effects of hypertensive disorders and vaginal bleeding were stronger for emergency CS. Oxytocin use, premature labour and suspected intrauterine death were associated with lower probability of an intrapartum CS.

**Table 3:** **Individual and institutional factors associated with elective, emergency and intrapartum caesarean sections (CS). Multivariable analysis**^**£**^

**Women characteristics**		**Adjusted OR (95% CI)**	
	**Elective CS (*n*=83 122)**	**Emergency CS (*n*=85 737)**	**Intrapartum CS (*n*=81 040)**
Age ≥35 years (vs. <35 years)	1.9 (1.7-2.2)	1.3 (1.2-1.5)	1.2 (1.1-1.3)
Multiple pregnancy (vs. singleton)	2.1 (1.7-2.6)	1.3 (1.1-1.6)	1.6 (1.5-1.8)
Nulliparous (vs. multiparous)	1.6 (1.4-1.8)	1.6 (1.5-1.8)	1.8 (1.7-1.8)
Previous caesarean section	19.2 (17.2-21.6)	5.5 (4.8-6.2)	8.9 (8.3-9.6)
Hypertensive disorders ^§^	2.2 (1.8-2.6)	7.7 (6.9-8.6)	1.1 (1.0-1.2)
Vaginal bleeding (near full term)		10.2 (8.9-11.6)	2.0 (1.8-2.2)
Premature rupture of membranes		3.9 (3.4-4.5)	2.2 (2.0-2.4)
Suspected cephalopelvic-disproportion			2.8 (2.0-4.0)
Referred from another facility		1.5 (1.3-1.6)	5.7 (5.4-6.0)
Oxytocin use			0.3 (0.3-0.4)
Premature labour			0.2 (0.2-0.3)
Suspected intrauterine death			0.3 (0.3-0.4)
**Hospital characteristics**			
Senegal (vs. Mali)		3.9 (2.6-6.1)	0.5 (0.4-0.7)
Adult intensive-care unit available		2.3 (1.5-3.5)	
Newborn care unit with incubators	1.6 (1.2-2.2)		
Neonatal resuscitation	1.7 (1.2-2.4)		
Medical staff configuration^*^			
Level I	1	1	1
Level II	2.0 (1.0-4.1)	0.9 (0.4-1.9)	0.9 (0.6-1.4)
Level III	4.8 (2.6-8.8)	1.5 (0.7-3.2)	1.0 (0.7-1.3)
Level IV	9.4 (5.1-17.1)	1.5 (0.7-3.5)	1.1 (0.8-1.6)
Anaesthetist 24h/day in hospital		2.7 (1.8-4.0)	

OR, Odd’s ratio; CI, confidence interval.

^£^ The analyses were conducted using logistic mixed models adjusted for the period. In the first model, elective CS were compared with all deliveries with a trial of labour; the institutional variables that were not included in the final model after a forward-stepwise procedure were: country, generator, adult intensive care unit, ultrasound services, urine culture, proteinuria, electronic foetal monitoring, anaesthetist 24h/day in hospital, maternal cardio-pulmonary resuscitation, at least one pediatrician and annual number of deliveries. In the second model, emergency CS were compared with all other deliveries; the institutional variables that were not included in the final model after a forward-stepwise procedure were: generator, proteinuria, glucose-tolerance test, urine culture, electronic foetal monitoring, neonatal resuscitation and maternal cardio-pulmonary resuscitation. In the third model, intrapartum CS were compared with all vaginal deliveries; the institutional variables that were not included in the final model after a forward-stepwise procedure were: high-risk consultation clinic, urine culture and at least one resident MD in training ^§^ Chronic hypertension, gestational hypertension, pre-eclampsia, eclampsia, HELLP syndrome; * Level I: Trained general practitioner, nurse-anaesthetist, ≤2 midwives; Level II: trained general practitioner, nurse-anaesthetist, ≥3 midwives; Level III: obstetrics specialist, nurseanaesthetist, ≥3 midwives; Level IV: obstetrics specialist, medical anaesthetist, ≥3 midwives.

Independently of individual risk factors, women had a 5- to 9-fold higher risk for elective CS in level III and IV hospitals compared to level I hospitals. Furthermore, elective CS was associated with the availability of neonatal-care services. The probability of an emergency CS was higher in facilities where there was an adult intensive-care unit and an anaesthetist on call 24 h/day, and was lower in facilities with >1 000 annual deliveries. Intrapartum CS rate was not influenced by institutional factors.

Independently of individual and institutional factors, the probability of emergency CS was higher in Senegal than in Mali, but intrapartum CS rate was lower in Senegal. For elective CS, institutional and individual factors that were included in the final model explained, respectively, 69% and 11% of the between-hospital variability (20% residual variability). For emergency CS, these proportions were, respectively, 53% and 33% (14% residual variability). For intrapartum CS, they were, respectively, 20% and 35% (45% residual variability).

Finally, we estimated that the expected proportion of low-risk women with elective CS varied from 0.07–0.3% for level I hospitals and from 0.6–2.6% for level IV hospitals (data not shown). For high-risk women, the proportions varied from 3–13% for level I hospitals, but were as high as 25–58% in level IV hospitals.

## Discussion

Two years after the implementation of free-CS policy in Senegal and Mali, we assessed the non-financial factors for women who had a CS in a referral hospital. The study confirmed the significant effect of well-known maternal risk factors on the mode of delivery. Adult intensive-care units, neonatal-care services, a 24h/day anaesthetist, workload and the configuration of the medical staff were independent institutional factors for CS.

CS rates were highly variable between facilities, ranging from 8–46%. As reported for other African countries, most caesarean deliveries were intrapartum, and maternal indications accounted for most of them (77%) [11,12,23]. Prolonged/obstructed labour or suspected cephalopelvic disproportion and previous caesarean section were the most frequent maternal indications. These results are in accordance with previous reports from the same and other sub-Saharan Africa countries. Interestingly, in 1998, a previous caesarean section was found to be the main indication for 6% of CS in all referral hospitals in Senegal [24], compared to 13% in the current study.

As expected, the following women’s characteristics were associated with a higher risk of intrapartum CS: age ≥35, nulliparity, multiple pregnancy, previous CS, vaginal bleeding, suspected cephalopelvic-disproportion and premature rupture of the membrane [11,16]. Oxytocin use was a protective factor for intrapartum CS, as shown by a recent meta-analysis on active management of labour [25]. Premature delivery and suspected intrauterine death were associated with a decreased risk of intrapartum CS for women in labour. In these women, vaginal delivery may have been favoured to reduce the risk of maternal complications associated with caesarean section.

We used data collected from most of the referral hospitals in Senegal and Mali. All deliveries were prospectively recorded and data quality was regularly controlled. Both the very large sample size (almost 90 000 deliveries) and the high number of CS allowed us to assess numerous factors with sufficient statistical power. The 41 included hospitals were representative of the existing health system in both countries, taking into account the variety of contexts (urban vs. rural) and the levels of care (primary vs. secondary referral health facilities). Our findings may be extrapolated to other referral hospitals in West Africa with similar recruitments and characteristics. However, more data on practices related to CS in all African countries is needed.

Our study has some limitations. First, diagnoses during the current pregnancy were recorded from open questions (e.g. vaginal bleeding near full term), and may have been reported differently among hospitals. Some misclassifications probably occurred by categorizing some exposed women (i.e. who had a condition) as non-exposed. If differential according to the mode of delivery, these misclassifications may have overestimated the level of association of corresponding individual factors. Second, the standardized inventory we used to assess available resources was not defined to assess 24h/day availability and real utilization of all the resources [16]. Some misclassifications probably occurred by categorizing some exposed hospitals (resource available but not used) as non-exposed, leading to possible underestimation of the corresponding Odds ratios. Thirdly, a large part of the between-hospital variability (45%) remained unexplained for intrapartum CS. This heterogeneity may be due to financial and medical practitioner factors that were not measured in our study. Financial barriers may be still present in some hospitals where the policy has not been fully implemented [7]. Younger physicians working alone in remote areas may have different practices than older physicians working in cities [9,26]. Taking this information into account would improve the performance of our predictive model, but would not affect the results dramatically.

Although numerous studies have assessed individual and institutional factors as predictors for CS [11-13,16,27,28], few have been conducted in SSA [18]. In a systematic review, Torloni et al. identified the main CS classifications used worldwide and analysed the advantages and deficiencies of each system [29]. Their results suggest that women-based classifications are the most appropriate system. Our findings confirm this finding for referral hospitals in low-income countries. The use of standard low-risk-patient definitions, based on a woman’s characteristics (i.e. age <35 years, multiparous, singleton pregnancy, no previous CS) and with no pathologies during pregnancy can facilitate auditing, analysis and comparison of CS rates across different settings. Our results also suggest that CS rates should be analysed according to available resources, workload and staff configurations. We recommend that analyses be performed according to the degree of urgency of CS as the effects of institutional factors are different for emergency ante-partum, intrapartum and elective CS.

## Conclusions

Our study has identified the main non-financial predictors for CS in a representative sample of referral hospitals in Senegal and Mali. We confirm that a woman-based CS classification system is appropriate in low-resources settings. The availability of intensive care, workload, staff configuration and the degree of urgency of the CS also need to be taken into account when analysing and comparing CS rates in this context. The use of single CS classification will facilitate international and local comparisons. Further studies are needed to evaluate the impact of rapidly changing practices related to CS on maternal and perinatal outcomes and to explain why more specialized staff perform more elective CS.

## Competing interests

The authors (VB, AD, MA, MT, LW and PF) declare that they have no competing interests.

## Authors’ contributions

AD, PF and MA participated in the design of the study. AD and PF were the principal investigators for the trial. AD and MT contributed to the implementation of the study. VB led the data analyses under the supervision of LW and MA. AD, MT, PF, LW and MA contributed to the interpretation of the results. The writing of the paper was led by VB and AD, with inputs from all other investigators. All authors read and approved the final manuscript.

## Pre-publication history

The pre-publication history for this paper can be accessed here:

http://www.biomedcentral.com/1471-2393/12/114/prepub

## Supplementary Material

Additional file 1 **Health facilities’ characteristics and available resources and services, by time period (*n* (%)).** Click here for file
