# Supplementary material for: Individual and institutional determinants of caesarean section in referral hospitals in Senegal and Mali: a cross-sectional epidemiological survey
Source: BMC Pregnancy Childbirth. 2012 Oct 22;12:114. doi: 10.1186/1471-2393-12-114 (PMC3534628; doi:10.1186/1471-2393-12-114)
Supplement: Additional file 1 — Health facilities’ characteristics and available resources and services, by time period (n (%)). [file 1471-2393-12-114-S1.doc]

Additional file 1.

|  | **Period 1**  **(*n*=41 hospitals)** | **Period 2**  **(*n*=41 hospitals)** |
| --- | --- | --- |
| **Health facilities’ characteristics** |  | |
| Country |  | |
| Mali | 21 (51) | |
| Senegal | 20 (49) | |
| Type^*^ |  | |
| Hospital in the capital | 12 (29) | |
| Regional hospital outside the capital | 14 (34) | |
| District hospital | 15 (37) | |
| Annual number of deliveries |  | |
| <1,000 | 13 (32) | |
| 1,000–3,000 | 22 (54) | |
| ≥3,000 | 6 (14) | |
| **Resources and services** |  |  |
| ***Basic services*** |  |  |
| Electricity | 40 (98) | 40 (98) |
| Generator | 38 (93) | 38(93) |
| Telephone | 39 (95) | 36 (88) |
| ***General medical services*** |  |  |
| Blood bank | 35 (85) | 34 (83) |
| Safe blood | 21 (51) | 22 (54) |
| Adult intensive care unit | 17 (41) | 17 (41) |
| Neonatal intensive care unit | 9 (22) | 10 (24) |
| High-risk pregnancy beds | 29 (71) | 29 (71) |
| Radiology department | 33 (80) | 35 (85) |
| Ultrasound services | 38 (93) | 38 (93) |
| Newborn care unit with incubators | 10 (24) | 10 (24) |
| High-risk consultation clinic | 35 (85) | 36 (88) |
| Medical clinics for referral in the same building | 37 (90) | 37 (90) |
| ***Screening tests*** |  |  |
| Proteinuria | 35 (85) | 34 (83) |
| Hepatitis B | 40 (98) | 40 (98) |
| Glucose-tolerance test | 37 (90) | 37 (90) |
| Pap smear | 3 (7) | 3 (7) |
| Urine culture | 23 (56) | 25 (61) |
| Alpha fetoprotein | 2 (5) | 2 (5) |
| Colposcopy | 7 (17) | 10 (24) |
| ***Anaesthesiology resources*** |  |  |
| Anaesthetist 24h/day in hospital | 16 (39) | 18 (44) |
| Anaesthetist on call, but outside hospital | 27 (66) | 25 (61) |
| Nurse-anaesthetist | 40 (98) | 40 (98) |
| ***Basic emergency obstetric services*** |  |  |
| Blood transfusion | 40 (98) | 40 (98) |
| Neonatal resuscitation | 29 (71) | 32 (78) |
| Maternal cardio-pulmonary resuscitation | 20 (49) | 22 (54) |
| ***Intrapartum care*** |  |  |
| Partograph | 39 (95) | 41 (100) |
| Staff skilled in: |  |  |
| Forceps extraction | 38 (93) | 38 (93) |
| Vacuum extraction | 33 (80) | 34 (83) |
| Electronic foetal monitoring | 10 (24) | 9 (22) |
| Foetal scalp pH | 1 (2) | 1 (2) |
| ***Human resources*** |  |  |
| Medical staff configuration |  |  |
| Trained general practitioner, nurse-anaesthetist, ≤2 midwives | 8 (19) | 5 (12) |
| Trained general practitioner, nurse-anaesthetist, ≥3 midwives | 5 (12) | 8 (19) |
| Obstetrics specialist, nurse-anaesthetist, ≥3 midwives | 15 (37) | 14 (34.5) |
| Obstetrics specialist, medical anaesthetist, ≥3 midwives | 13 (32) | 14 (34.5) |
| At least one resident MD in training | 25 (61) | 21 (51) |
| ***Academic resources and clinical protocols*** |  |  |
| Medical library | 6 (15) | 10 (24) |
| Formal protocols for: |  |  |
| Antenatal care | 34 (83) | 33 (80) |
| Intrapartum care | 35 (85) | 34 (83) |
| Post-partum care | 35 (85) | 34 (83) |
| Neonatal care | 36 (88) | 31 (76) |
| Other medical/nursing protocols | 37 (90) | 37 (90) |
| Continuous medical education programme | 32 (78) | 38 (93) |

Period 1: October 2007–March 2008; Period 2: April 2008–October 2008.

^*^ In Senegal, 6 hospitals were located in Dakar, 10 were regional hospitals and 4 were districts hospitals. In Mali, 6 hospitals were located in Bamako, 4 were regional hospitals and 11 were districts hospitals.
